# Artificial neural network model for predicting the bioavailability of tacrolimus in patients with renal transplantation

**DOI:** 10.1371/journal.pone.0191921

**Published:** 2018-04-05

**Authors:** Kalluri Thishya, Kiran Kumar Vattam, Shaik Mohammad Naushad, Shree Bhushan Raju, Vijay Kumar Kutala

**Affiliations:** 1 Departments of Clinical Pharmacology and Therapeutics, Nizam's Institute of Medical Sciences Hyderabad, Telangana, India; 2 Sandor Lifesciences Pvt Ltd, Hyderabad, Telangana, India; 3 Department of Nephrology, Nizam's Institute of Medical Sciences, Hyderabad, Telanagana, India; University of Toledo, UNITED STATES

## Abstract

The objective of the current study was to explore the role of ABCB1 and CYP3A5 genetic polymorphisms in predicting the bioavailability of tacrolimus and the risk for post-transplant diabetes. Artificial neural network (ANN) and logistic regression (LR) models were used to predict the bioavailability of tacrolimus and risk for post-transplant diabetes, respectively. The five-fold cross-validation of ANN model showed good correlation with the experimental data of bioavailability (r^2^ = 0.93–0.96). Younger age, male gender, optimal body mass index were shown to exhibit lower bioavailability of tacrolimus. ABCB1 1236 C>T and 2677G>T/A showed inverse association while CYP3A5*3 showed a positive association with the bioavailability of tacrolimus. Gender bias was observed in the association with ABCB1 3435 C>T polymorphism. CYP3A5*3 was shown to interact synergistically in increasing the bioavailability in combination with ABCB1 1236 TT or 2677GG genotypes. LR model showed an independent association of ABCB1 2677 G>T/A with post transplant diabetes (OR: 4.83, 95% CI: 1.22–19.03). Multifactor dimensionality reduction analysis (MDR) revealed that synergistic interactions between CYP3A5*3 and ABCB1 2677 G>T/A as the determinants of risk for post-transplant diabetes. To conclude, the ANN and MDR models explore both individual and synergistic effects of variables in modulating the bioavailability of tacrolimus and risk for post-transplant diabetes.

## Introduction

Tacrolimus is an immunosuppressive agent that is prescribed to prevent acute rejection following solid organ transplantation such as kidney, liver, and heart transplantations [1–3]. It is widely used agent, however, characterized by narrow therapeutic index and high inter-individual variability in dose requirement necessitating frequent therapeutic drug monitoring to prevent acute rejection or renal toxicity [3]. The trough concentrations of tacrolimus are known to influence the clinical outcome, i.e., prevention of organ rejection [4], whereas high trough concentrations have been reported to cause toxicity [5, 6]. Acute rejection is a major causal factor for long-term graft outcome, and it commonly occurs during the immediate post-transplantation [7]. Several factors such as age, gender, ethnicity, body-weight, serum creatinine, albumin, haematocrit, days after transplantation and the use of concomitant drugs will greatly influence the tacrolimus pharmacokinetics [8, 9].

Several studies have examined the role of genetic polymorphisms in CYP3A5 and ABCB1 influencing tacrolimus concentration as well and its clinical outcome [10–13]. CYP3A5*1 is the wild-type allele is associated with enzyme activity, while CYP3A5*3 is the most common allele that causes splice defect and resulting in non-functional protein and thus requiring a high dose of tacrolimus for the patients [14]. Many studies have investigated the association of CYP3A5*1/*3 polymorphism and tacrolimus pharmacokinetics in renal transplantation [11, 12, 15, 16]. A significant association between the CYP3A5*1/*3 variant allele and tacrolimus pharmacokinetics was well established [17]. The dose required to reach the therapeutic concentration range was estimated to be twice as much as in carriers of at least one active CYP3A5 (AA/AG) allele than in non-carriers (GG) [13].

The role of polymorphisms in drug transporter, ABCB1 gene on trough concentrations of tacrolimus yielded mixed results. One study described the association of ABCB1 polymorphisms with the dose-adjusted tacrolimus trough concentrations during the early period after transplantation [18]. In another study, the ABCB1 2677G>T and ABCB1 3435C>T polymorphisms were found to be associated with lower dose-adjusted levels of tacrolimus and were at increased risk of allograft rejection [19], whereas in another study, patients expressing the wild-type ABCB13435CC genotype showed up to 40% lower concentration/dose ratios compared with patients carrying variant alleles [20]. Several studies have found no association between the tacrolimus trough concentration and ABCB1 polymorphisms [21–24]. Patients with wild ABCB1 haplotype reported to have lower dose-normalized tacrolimus concentrations compared with patients carrying the variant copy of this gene [25].

Recent studies have indicated the role of acquired as well as genetic factors which are associated with tacrolimus efficacy. For the precise dosing of tacrolimus, a number of algorithms have been developed by using clinical and genetic factors namely CYP3A5 and ABCB1 [26–32]. A randomized clinical trial, demonstrated the importance of CYP3A5 genotype-guided tacrolimus dosing to achieve target tacrolimus trough (C_0_) levels after three days of tacrolimus treatment [27]. Further, in the same study, they observed that patients took less time to reach their target concentration with fewer dose modifications [27]. In a recent study on CYP3A4 and CYP3A5 genotypes revealed 56–59% variability in tacrolimus dose requirement and clearance [26]. Low exposure to tacrolimus during early post-transplant days showed increased risk of acute rejection [29]. The acute rejection is an early event with the greater number of occurrence within the first 12 weeks and lower incidences between 16 to 24-weeks of the post-transplant period. Hence, any prediction model that predicts tacrolimus dose before the transplantation by utilizing both acquired and genetic factors, to reach the therapeutic dose faster with minimum dose adjustments may benefit to the patients. Hence, the present study was carried to develop an algorithm for the prediction of tacrolimus dose by using acquired and genetic factors in kidney transplant cases from south Indian population.

## Materials and methods

### Recruitment of subjects

This is a prospective study involving 129 patients undergoing renal transplantation recruited from the department of Nephrology, Nizam’s Institute of Medical Sciences (NIMS), Hyderabad, India. Demographic details like age, gender, weight, height, biochemical and hematological investigations at the time of sample collection, clinical history and concomitant medications were recorded. The clinical outcomes considered are an occurrence of acute rejection, loss of renal function and serious infections during six months of the study period. Patients with impaired liver function, combined organ transplantation were excluded from the study. The study was approved by the ethics committee of Nizam’s Institute of Medical Sciences (NIMS), EC/NIMS/1379/2013 Hyderabad, India. Written informed consent was obtained from all the subjects.

### Immunosuppressive regimen

All the patients received the triple immunosuppressive regimen namely tacrolimus, mycophenolate mofetil (MMF) and steroids. One day prior to transplant, all the patients received a daily oral treatment with tacrolimus at a dose of 0.15 mg/kg administered in two divided doses. The dose was then adjusted according to C0 levels: 5–15 and 5–10 ng/ml during early and late post-transplant phases, respectively. MMF was given at a dose of 720 mg twice daily and 20 mg of wysolone per day.

### Sample collection and measurement of tacrolimus (C0)

To determine the trough concentrations of tacrolimus, blood samples were collected from 54 patients on day 1, 3, 5, 10, 15, 30, 45, 60 and 90 day post-transplantation before taking tacrolimus on that particular day. Tacrolimus concentrations were determined by commercially available kit (Roche Diagnostics) by using fully automated immunology analyzer (Roche Cobas E411). Appropriate quality control samples were also used for the quality check. The sensitivity of this kit was 1 ng/ml. Dose-normalized tacrolimus concentrations (C0: dose, ngml^-1^ per mg/day per kg body weight) were calculated by dividing the tacrolimus trough concentration (Co, ngml^-1^) by the daily dose adjust for body weight.

### Genetic analysis

Whole blood samples were collected in EDTA vacutainers and the buffycoat was used for genomic DNA isolation by using standard phenol-chloroform extraction method. CYP3A5*3 (6986A>G), ABCB1 1236 C>T and ABCB1 2677 G>T/C alleles were determined by using Sanger’s sequencing, outsourced to Bioserve Technologies Pvt. Ltd. Hyderabad, India on ABI Prism 3730XL Genetic Analyzer (Applied Biosystems). FinchTV software was used to visualize the sequencing chromatograms and genotypes were noted comparing with reference sequences. ABCB1 3435 C>T polymorphism was analyzed using PCR-RFLP method. The PCR products were digested with Mbo I restriction enzyme. The Mbo I enzyme digestion of the 197 bp PCR amplicon produces 158 bp and 39 bp product for the mutant type allele, but fails to cleave the 197 bp fragment with wild type allele. Heterozygous allele produces 197 bp, 158 bp and 39 bp bands [33,34]. All the primers used for genotyping were represented in Table 1. Sanger’s sequencing was done to confirm the genotyping in thirty percent of the samples and found 100% concordant.

**Table 1:** Primers used for genetic analysis.

S No.	rs number	Gene	Nucleotide change	Primer
1	rs776746	CYP3A5*3	A>G	F:5'-CTGCCCTTGCAGCATTTAGT-3' R:5'-CAGCACAGGGAGTTGACCTT-3'
2	rs1128503	ABCB1 1236	T>C	F:5'-CCTGACTCACCACACCAATG-3' R:5'-TCACTTTATCCAGCTCTCCACA-3'
3	rs2032582	ABCB1 2677	G>A/T	F:5'-AAAGTGGGGAGGAAGGAAGA-3' R:5'- TCAGCATTCTGAAGTCATGGA-3'
4	rs1045642	ABCB1 3435	T>C	F:5`-TCTTTTCAGCTGCTTGATGG-3' R:5`-AAGGCATGTATGTTGGCCTC-3'

Genotyping of CYP3A5*3 ABCB1 1236 ABCB1 2677 was done by Sanger’s sequencing whereas the ABCB1 3435 was done by PCR-RFLP method

### Artificial neural network based algorithm development

**Fig 1:**
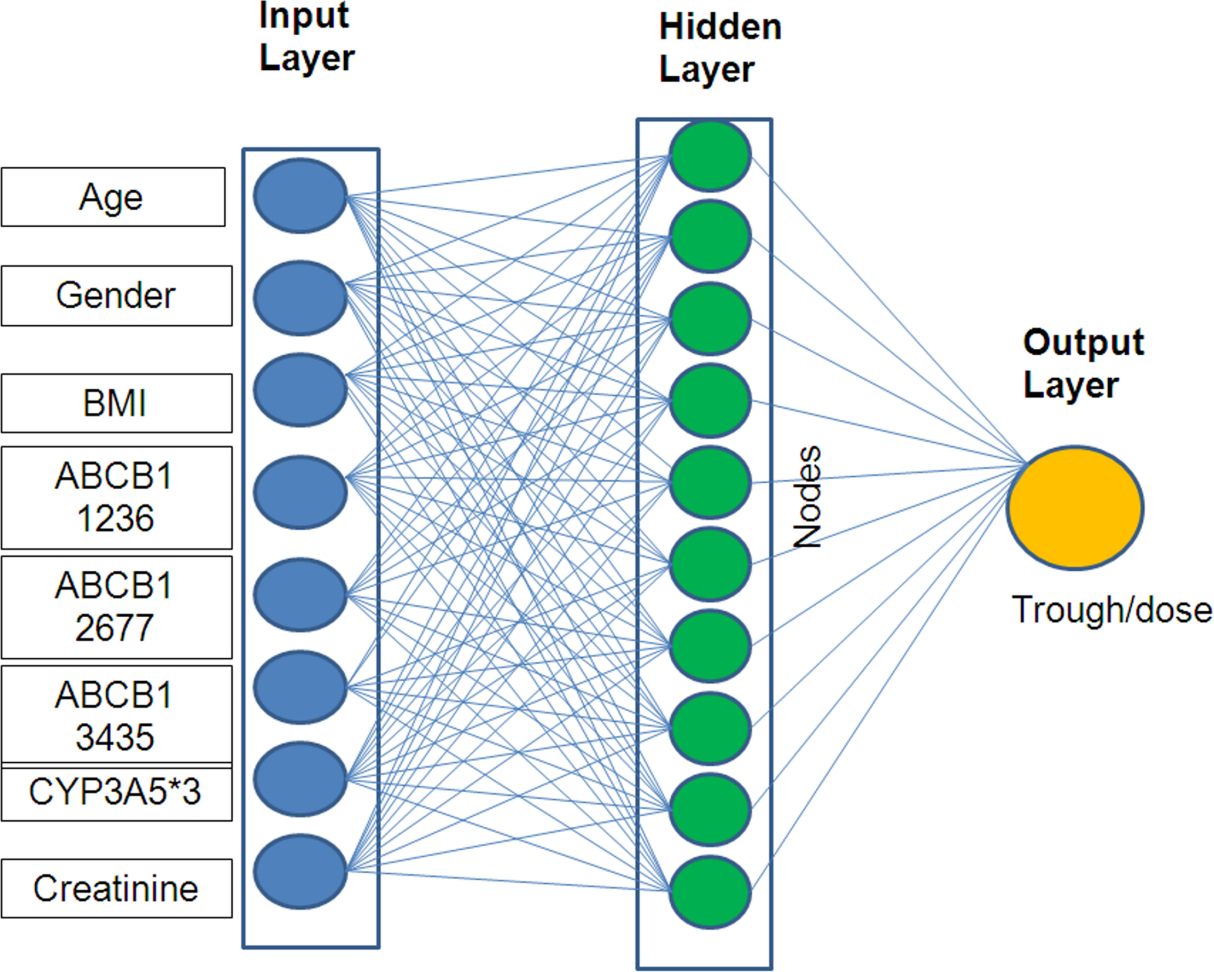
Schematic representation of artificial neural network architecture. The artificial neural network model is comprised of three layers namely input layer, hidden layer and output layer. The input layer has age, gender, body mass index, CYP3A5*3, ABCB1 1236 C>T, ABCB1 2677 G>A/T, ABCB1 3435 C>T and creatinine as input variables. The hidden layer is optimized to have ten nodes whose weights are based on Bayesian approximation. The output variable is bioavailability in terms of ratio of plasma concentration with oral dose of tacrolimus.

We have used Bayesian averaging or error-correcting output coding, bagging and boosting as the basis of the model. The computational website www.bigml.com was used for modeling. The code is supplied as S1 Table. As illustrated in **Fig 1**, the ANN architecture was comprised of three layers i.e. an input layer, a hidden layer and an output layer. The input variables were age, gender, body mass index, creatinine, CYP3A5*3 (6986A>G), ABCB1 3435 C>T, ABCB1 1236 C>T and ABCB1 2677 G>T/C. The number of nodes in the hidden layer was optimized to ten based on the root mean square error (RMSE) statistics. The output variable in this model is the bioavailability depicted in terms of the ratio of plasma tacrolimus concentration with the oral dose. Experimental data ranges from input and output variables were tabulated as **Table 2**. In order to have better validation, we have divided the data into 5 subsets by retaining 20% of the data at a time and performed 5-fold cross-validation of our model. Mean square error and regression coefficient were used as a measure to assess network performance. For this approach, genotype data was computed as 0, 1 and 2 based on the number of variant alleles. For the development of the model, we have used the computational website www.bigml.com.

**Table 2:** The input and output variables of the developed ANN model.

Input variable	Range	Mean ± SD
Age (yr)	17 – 61	32.4 ± 9.8
Body mass index (kgm^-2^)	11.7 – 30.1	20.5 ± 3.5
Male: Female	108: 28	
CYP3A5 *1/*1:*1/*3:*3/*3	26:58:52	
ABCB1 1236 CC:CT:TT	40:59:37	
ABCB1 2677 GG:GA/GT:TT/AT/AA	15:51:70	
ABCB1 3435 CC:CT:TT	31:65:40	
Output variable	Range	Mean ± SD
Trough/dose	0.31-32.40	3.45 ± 4.45

### Statistical analysis

To compute genotype data, 0 and 1 were used depending on the number of variant alleles. All the SNPs were checked for deviation from Hardy–Weinberg equilibrium using χ2—test between the observed and expected frequencies. In derivation and validation cohort samples, Student’s t-test was done to assess statistically significant differences among the continuous variables. Using Epimax calculator, the rate of overestimation and rate of underestimation were calculated. A “p” value of <0.05 was considered as significant. Logistic regression analysis was carried out to evaluate independent association of demographic and genetic variables with post-transplant diabetes. Multifactor dimensionality reduction analysis was carried out to explore gene-gene interactions that increase the risk for post-transplant diabetes.

## Results

In the present study, we included 129 renal transplant patients. There were 102 men and 27 women. The mean age was 34.2 ± 11.44 yrs; mean body mass index was 20.5 ± 3.5 kgm^-2^. At the time of transplantation, hematological and biochemical parameters including liver function tests were within normal limits (**Table 3**). After 3 months of post-transplant, we observed the acute rejection in 5.76% of the patients, viral infections (CMV, BK, HCV) in 2.88% of patients, skin infection was seen in 0.96% of patients and repeated infections more than once was observed in 5.76% of patients (**Table 3**).

**Table 3:** Demographic characteristics of studied subjects (n = 129).

Parameter	Result
Male:Female	102:27
Age (years)	34.2±11.4
Body mass index (kgm^-2^)	54.8±12.0
Related Donor	71.16%
**Other diagnosed diseases:**	
Diabetes	26.40%
Thyroid	12.00%
Hypertension	60.14%
**Clinical parameters:**	
SGOT (U/L)	16.8±6.28
SGPT (U/L)	12.7±4.3
Total Protein (g/ml)	6.8±0.58
Albumin (g/dl)	5.2±6.6
T.Bilirubin (mg/dl)	0.42±0.17
Random blood glucose (mg/dl)	98.16±17.85
Urea (mg/dl)	74.75+47.44
Serum Creatinine (mg/dl)	1.16±0.28
Total leukocyte count (cells/mm^3^)	7896±2692
**Other drugs:**	
Mycophenolate (0.3–1.5 g/day)	98.1%
Azathioprine (3–5 mg/kg/day)	1.9%
**Adverse effects:**	
Acute rejection	5.76%
Viral infections(CMV, BK, HCV)	2.88%
Skin infection	0.96%
Repeat infections more than once	5.76%

All the patients were administered tacrolimus either once or twice daily. Additionally 98.1% received MMF (0.3–1.15 g/day) or 1.9% of patients received azathioprine (3–5 mg/day). The mean tacrolimus dose administered one day prior to transplant was 5.2 ± 1.2 mg/day, day 3 was, 5.29 ± 1.6 mg, day 30 was 4.24 ± 1.04 (p<0.0001); and on day 60 was 3.04 ± 1.5 g/day (p<0.001) (**Table 4**). Tacrolimus trough concentrations in 54 patients at predetermined days (486 samples) obtained during oral administration in the first 3 months post-transplantation. The initial tacrolimus dosing was based on individual’s body weight and subsequent doses were adjusted based on trough concentrations i.e., 5–15 ng/ml were targeted in the first 3 months and of 5–10 ng/ml in the subsequent 3–6 months of post- transplantation. The trough concentrations were determined on day 1, 3, 5, 10, 15, 30, 45, 60, and 90 after transplantation. As shown in Table 4, the mean trough concentrations on day 3, 30 and 60 was 11.1 ± 6.71, 6.06 ± 3.94 ng/ml (p<0001) and 4.46 ± 2.41ng/ml (p<0.0001) respectively. The dose-adjusted trough concentration ratio on day 3, 30 and 60 after transplantation was 120.7 ± 127.3, 77.54 ± 44.2 (p<0.001), and 94.6 ± 69.8 respectively. The percentage of patients within therapeutic range of tacrolimus (5–15 ng/ml) on day 3, 30 and 60 was 57.89, 48.65 and 34.8 percentage respectively, whereas below therapeutic range was 18.42, 48.65 and 65.52% and the percentage above therapeutic range was 23.68, 2.7 and 0% respectively (**Table 4**).

**Table 4:** Pharmacokinetics parameters in transplant cases.

Parameter	Day 3 (n = 54)	Day 30 (n = 54)	Day 60 (n = 54)
**Tacrolimus dose (mg/day)**	5.29±1.6	4.24±1.04*	3.04±1.5**
**Tacrolimus trough (C**_**0**_**) (ng/ml)**	11.1±6.71	6.06±3.94*	4.46±2.41***
**Log C**_**0**_ **/D**	120.7±127.3	77.54±44.2*	94.6±69.8
**C**_**0**_ **<therapeutic range (%)**	18.42	48.65	65.52
**C**_**0**_ **within therapeutic range (%)**	57.89	48.65	34.48
**C**_**0**_ **>therapeutic range (%)**	23.68	2.7	0

Log C_0_/D, log normalized trough/dose; therapeutic range: 5–15 ng/ml

*p<0.0001 vs day 3

**p<0.0001 vs day 30

***P<0.0001 vs day 30

**Fig 2:**
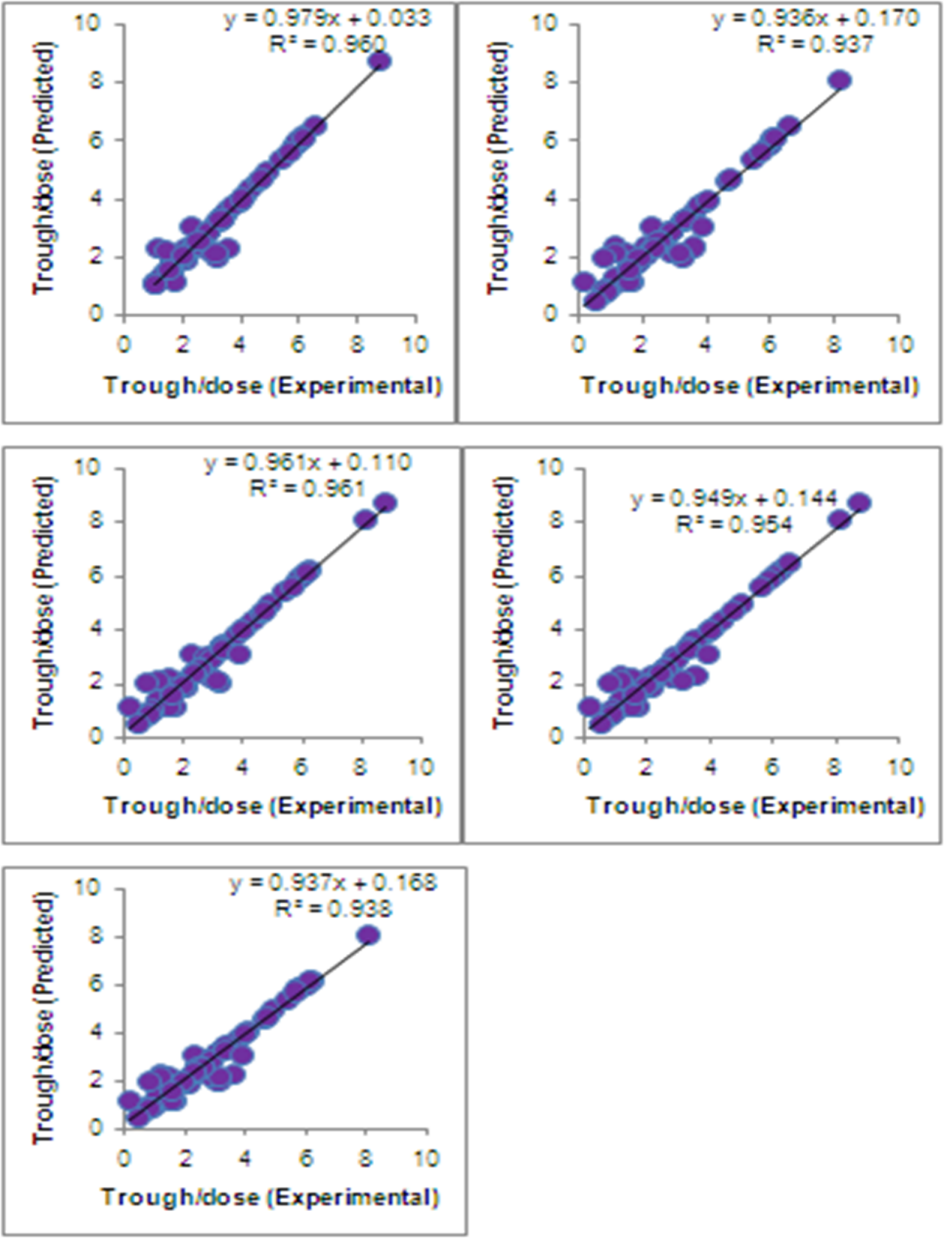
Cross-validation of ANN model. Five fold-cross validation of ANN model showed good agreement between experimental vs predicted trough/dose ratio (r^2^ = 0.94 to 0.96).

**Fig 3:**
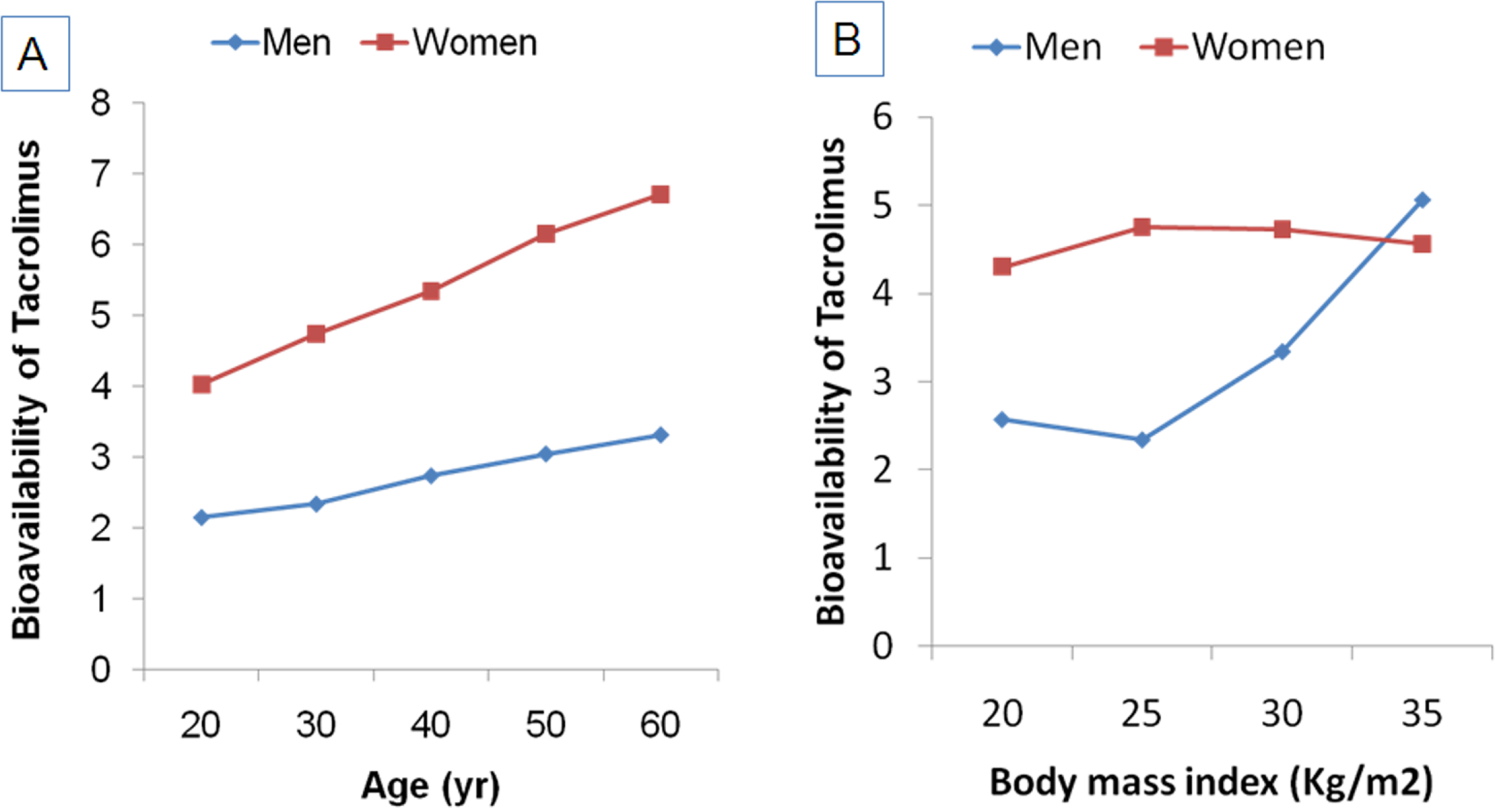
Impact of age, gender and BMI on the bioavailability of tacrolimus. ANN simulation illustrating (A) Age-dependent changes in bioavailability of tacrolimus in men and women; (B) Bioavailability of tacrolimus in men and women according to BMI.

All the polymorphism tested were found to be in accordance with Hardy-Weinberg equilibrium (HWE p>0.05). As shown in **Fig 2**, the five-fold cross-validation of ANN model (https://bigml.com/dashboard/model/5a30b752af447f1798000c61) of bioavailability of tacrolimus showed good agreement with the experimental data (r^2^ = 0.94 to 0.96). As shown in **Fig 3**, the ANN simulations of the effect of age and gender on the tacrolimus bioavailability indicate that women have more bioavailability of tacrolimus than men. With an increase in age, the bioavailability was shown to increase in both the genders. On the other hand, in men, the higher bioavailability of tacrolimus was observed in overweight and obese subjects. In women, no significant impact of BMI on bioavailability of tacrolimus was observed **(Fig 3A & 3B)**

**Fig 4:**
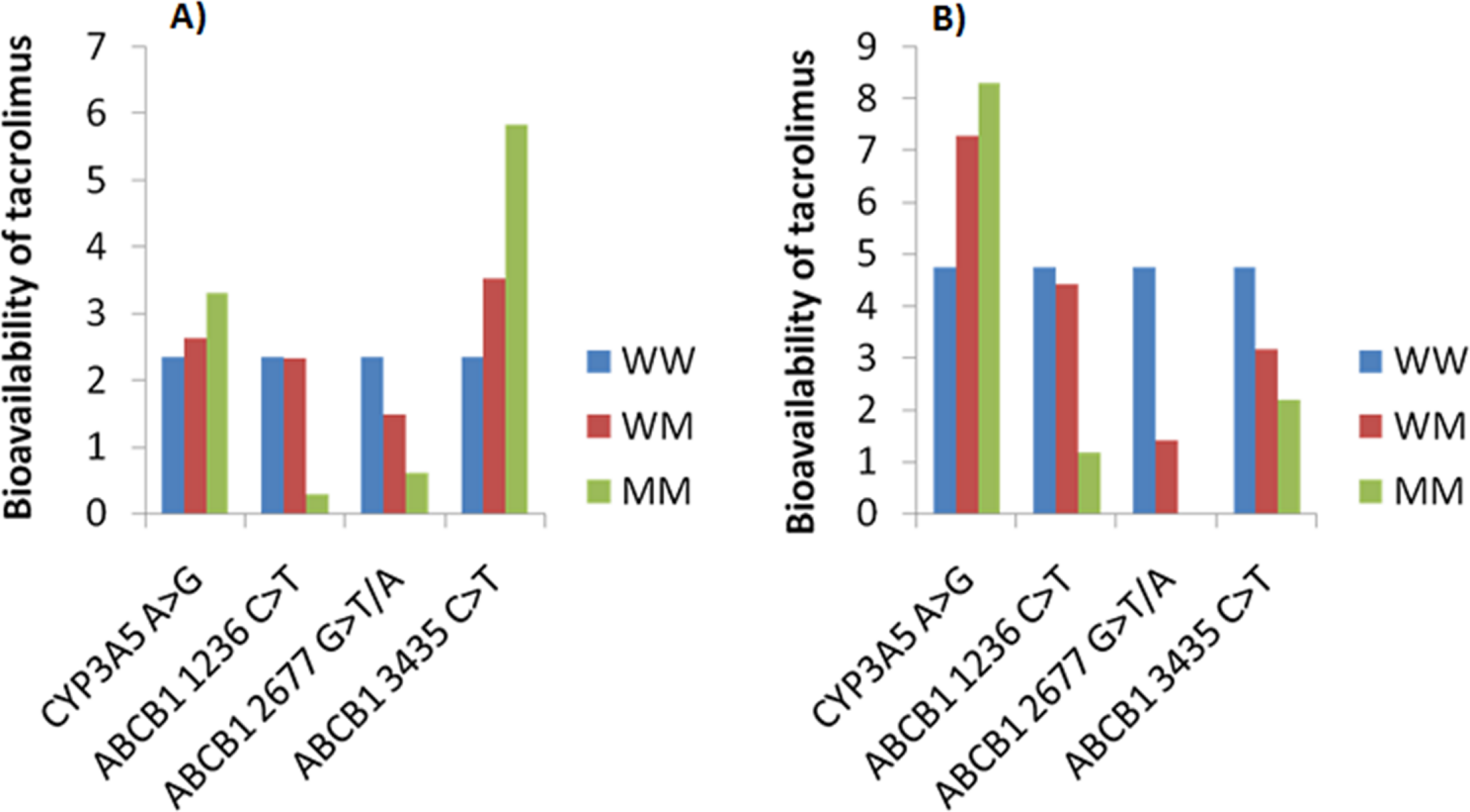
Impact of CYP3A5 and ABCB1 genotypes on the bioavailability of tacrolimus. ANN simulations depicting genotype based association of tacrolimus bioavailability in (A) men; (B) women.

The ANN simulations depicting genotype based association of tacrolimus bioavailability in men and women are shown in **Fig 4**. In men, the bioavailability of tacrolimus found to be high in patients carrying mutant allele in CYP3A5 and ABCB1 3435C>T, whereas lower bioavailability of tacrolimus was observed in patients carrying mutant allele in ABCB1 1236C>T and 2677 G>T/A (**Fig 4A**). On the other hand, in women, the bioavailability of tacrolimus was found to be high in patients carrying mutant allele in CYP3A5 where as lower bioavailability of tacrolimus was observed in patients carrying mutant allele in ABCB1 3435C>T, 1236C>T and 2677 G>T/A **(Fig 4B).**

**Fig 5:**
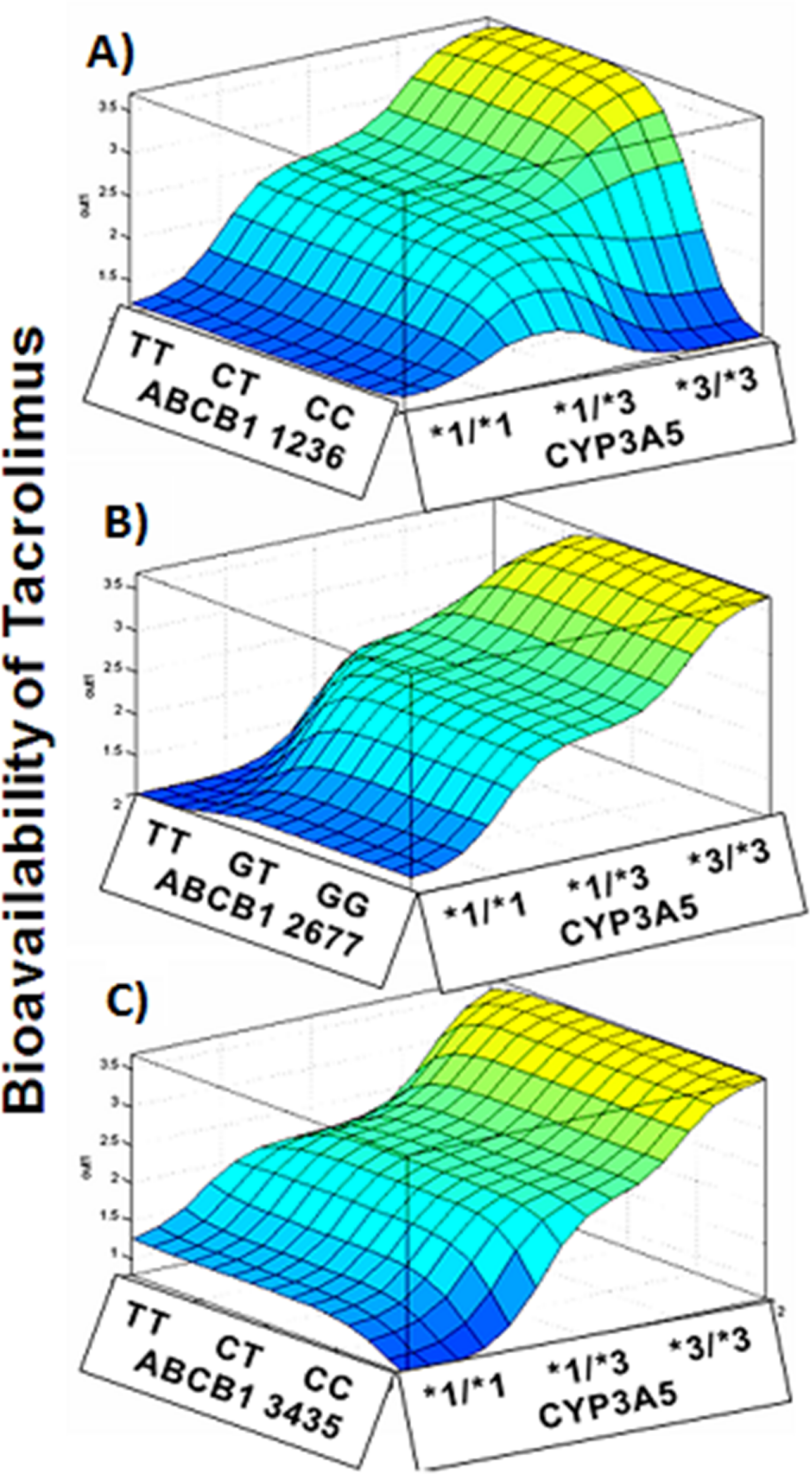
Gene-gene interactions modulating the tacrolimus bioavailability. ANN simulations depicting gene-gene interactions in modulating tacrolimus bioavailability. (A) CYP3A5*3 -ABCB1 1236 C>T; (B) CYP3A5-ABCB1 2677 G>T/A; (C) CYP3A5-ABCB1 3435 C>T.

**Fig 6:**
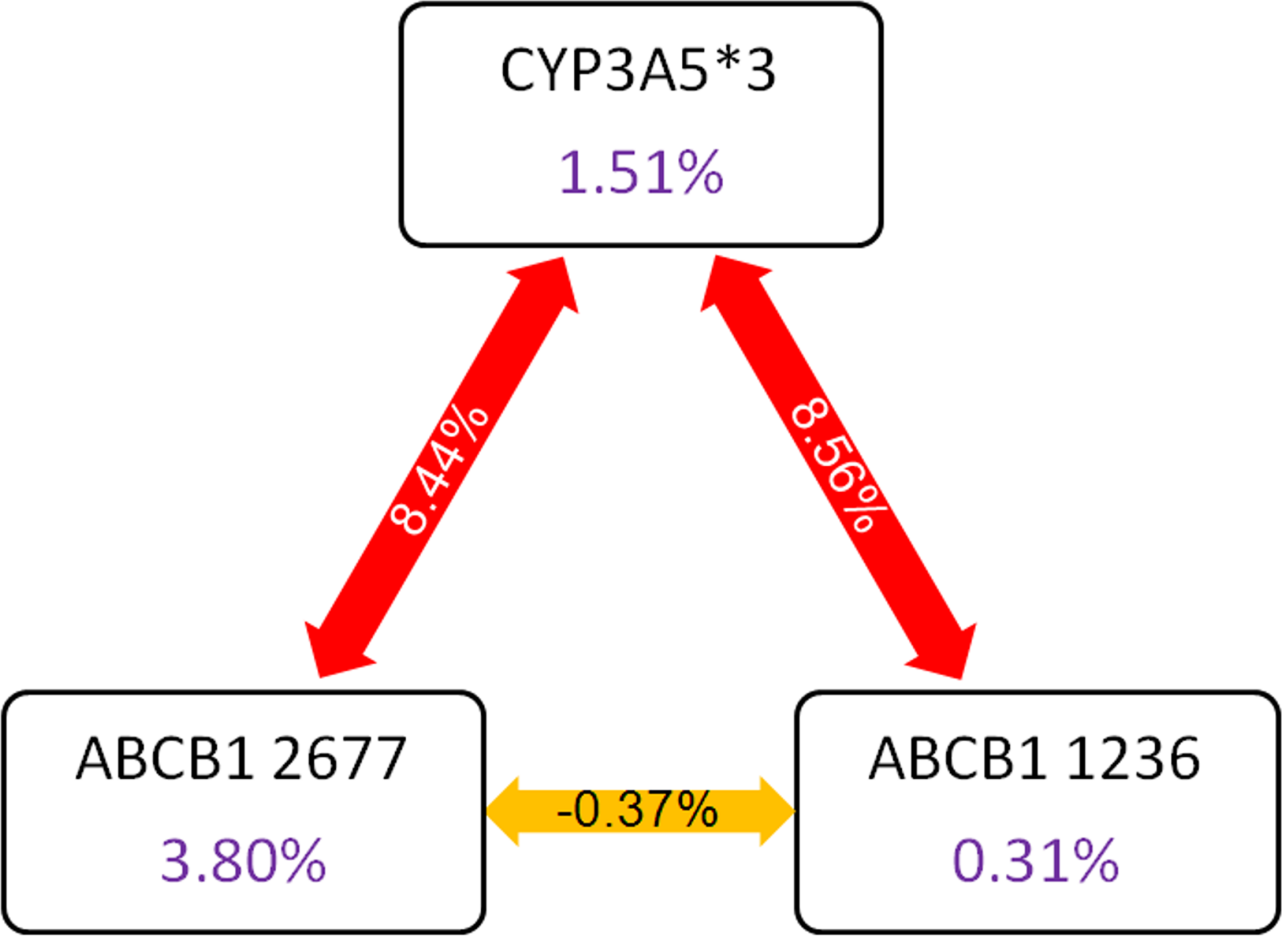
Frutcherman-Rheingold plot of multifactor dimensionality reduction analysis. Multifactor dimensionality reduction analysis revealed that CYP3A5*3 (X1) interacts strongly with ABCB1 1236 (X2) and ABCB1 2677 (X3) variants in influencing bioavailability of tacrolimus.

The ANN simulations showing gene-gene interactions in modulating tacrolimus bioavailability is depicted in **Fig 5.** The bioavailability of tacrolimus was found to be highest in subjects with CYP3A5 *3/*3/ABCB1 1236 TT combined genotype **(Fig 5A).** Subjects with CYP3A5*3/*3/ABCB1 2677 GG genotype exhibited higher bioavailability of tacrolimus **(Fig 5B).** ABCB1 3435 C>T polymorphism increases the bioavailability of tacrolimus. The presence of CYP3A5 *3/*3 synergistically increases the bioavailability further **(Fig 5C).** The intestinal absorption of tacrolimus was shown to be impaired in subjects harboring ABCB1 1236 C>T and 2677 G>T/A and 3436 C>T allelic variants. The CYP3A5*3 polymorphism was shown to have a positive association with the bioavailability. As shown in **Fig 6**, CYP3A5*3 variant interacts with variants of ABCB1 1236 and ABCB1 2677 to modulate the bioavailability of tacrolimus.

The acute rejection or dysfunction of the graft kidney was observed in 5.76% of the cases in the early post-transplantation phase. All these cases were found to have CYP3A5 mutant homozygous and heterozygous genotypes, whereas homozygous wild (AA), normal metabolizer, had no observed cases of rejection. The correlation of the number of dose changes with that of genotype AA genotype carriers have reached therapeutic range within 10 days of post transplantation and is associated with frequent dose changes (26.9%). Whereas AG (40.1%) and GG (32.93%) genotype carriers required 60 days of post transplantation with regular intervals of dose adjustments to reach targeted therapeutic range.

When the genotype related to slow metabolism (GG) CYP3A5 associated with T allele of ABCB1 gene (rs1128503, rs203258, rs1045642) in heterozygous or homozygous conditions (CT & TT) requires a higher number of dose adjustments. On average there were 21.74% allied with regular dose changes for first 10 days is observed to be normal metabolizers. The AG genotype had 39.2% and GG genotype 39.2% of dose changes after the 10^th^ day of tacrolimus treatment. The CT and TT genotype counts together to 47.53% while CC counts for 10.3% and other GG, GTand GA totally account to 6.5% dose changes.

As shown in **Table 5**, logistic regression analysis revealed the independent association of ABCB1 2677 G>T/A with post-transplant diabetes. As shown in **Fig 5**, multifactor dimensionality reduction analysis confirmed ABCB1 2677 G>T/A as the major determinant of post-transplant diabetes, which is having strong interaction with CYP3A5*3 polymorphism.

**Table 5:** Logistic regression analysis showing the impact of demographic and genetic variables contributing to post transplant diabetes.

Variable	Odds ratio	95% CI	P value
Age	1.02	0.95–1.09	0.59
Gender	1.81	0.33–10.07	0.50
Body mass index	1.06	0.86–1.30	0.61
CYP3A5*3	1.73	0.70–4.28	0.23
ABCB1 1236 C>T	2.59	0.79–8.50	0.12
**ABCB1 2677 G>T/A**	**4.83**	**1.22–19.03**	**0.02**
ABCB1 3435 C>T	0.86	0.34–2.19	0.76

## Discussion

The maintenance of renal graft cases that are on tacrolimus is challenging to the clinicians due to the narrow therapeutic index and wide inter-individual variability. Tacrolimus concentrations fluctuate greatly during the immediate post-transplant period and may be the causative factor for the increased rate of rejection and graft loss. Many studies have confirmed that CYP3A5 polymorphisms have a major influence on the pharmacokinetics of tacrolimus [35,36]. Patients who are homozygous for the CYP3A5*3 allele found to have lower dose requirements and higher trough levels of tacrolimus after transplantation, as well as lower clearance than patients expressing the CYP3A5*1 allele [23–29]. In the present study, we found that patients carrying at least one CYP3A5*1 allele are associated with decreased bioavailability of tacrolimus than patients carrying CYP3A5*3 allele, and this observation was seen even up to 4 weeks after the transplantation. A similar observation was also demonstrated in several studies showing transplant patients with at least one CYP3A5*1 allele had significantly higher tacrolimus dose requirements and lower trough drug levels than CYP3A5*3 homozygotes [20, 37].

Renal transplant recipients carrying CYP3A5*3 allele have lower dose requirement of tacrolimus when measured on 30, 90 and 180 days of post-transplantation as compared to CYP3A5*1 and CYP3A5*1/*3 [38]. In addition, several studies on CYP3A5*1 have demonstrated two-fold higher tacrolimus dose requirement to achieve the target drug levels [36–39]. In a prospective study based on the CYP3A5 genotype, the rapid achievement of target trough levels was observed in renal transplant recipients with fewer successive dose modifications prior to the transplantation [27].

In the current study, the acute rejection or dysfunction of the graft kidney was observed in 5.76% of the cases. This was observed in the early post-transplantation phase in patients carrying CYP3A5*3 and *1/3 genotype, but not observed in homozygous wild (AA) carriers. In a study, it was found that low tacrolimus troughs in the early post-transplant period have been associated with a higher rate of acute rejection [19]. The mean trough concentrations in the first-week post-transplant have shown to be significantly different between the patients with acute rejection and without rejection [28, 29].

Studies on ABCB1 polymorphisms have reported the association between P-glycoprotein expression and function with tacrolimus pharmacokinetics [11–13]. However, contradictory results have been reported regarding the effect of ABCB1 polymorphisms on tacrolimus kinetics and efficacy in organ transplantations [21–24]. In a study by Wang et al., found an association between the ABCB1 haplotype and blood tacrolimus concentration [32]. In another study on renal transplants, patients with the wild-type ABCB1 genotype tend to have more stable tacrolimus concentrations i.e., within the therapeutic range, during the 90 days after the transplantation [40]. Whereas patients carrying mutant alleles, showed increased tacrolimus concentrations due to decreased elimination capacity, over 60% [40]. In addition to the above study, wild-type ABCB1-3435CC genotype showed lower concentration/dose ratios compared with patients carrying variant genotype [20]. In the present study, we also observed that ABCB1 3435 variant genotype TT showed higher tacrolimus levels as compared to CT and CC genotypes. On the other hand, ABCB1 2677 and ABCB1 1236 polymorphism did not showed an association with the tacrolimus concentration.

Although there are no population-specific pharmacogenomic algorithms for Indians in predicting tacrolimus stable dose or bioavailability, however, certain polymorphisms were studied in renal transplant cases. Study by Singh et al, found that the dose-adjusted tacrolimus and cyclosporine levels were significantly lower in CYP3A5 expressers for cyclosporine and tacrolimus as compared to the non-expressers [41]. In the same study, they observed that CYP3A5 non-expresser genotype was associated with reduced risk for allograft rejection. Similarly, studies with ABCB1 polymorphisms, patients carrying wild-type at ABCB1 2677G>T and 3435C>T were associated with lower dose-adjusted levels and thereby were at increased risk of allograft rejection [42]. Chandel et al, studied the CYP3A5*1/*3 genotype influencing the tacrolimus blood concentrations in response to metabolic inhibition by ketoconazole [43]. A number of algorithms containing clinical and/or pharmacogenomic factors have been constructed to predict tacrolimus dose [26–33]. In our previous studies, we have developed and validated the algorithms for the precise prediction of warfarin dose [44, 45]. In the current study, with age, gender, BMI, CYP3A5*3 (6986A>G), ABCB1 1236 C>T and ABCB1 2677 G>T/C genotypes using ANN model; we have developed an algorithm for the prediction of bioavailability of tacrolimus. The tacrolimus-bioavailability model explained 86% of total variability in the tacrolimus absorption and metabolism. The other contributing factors could be albumin, hematocrit and liver function, that might influence during initial transplantation days [26].

In a prospective study on Caucasian kidney transplant patients, the effect of genotype-guided tacrolimus dosing versus body weight-based dosing have indicated that genotype-guided group had a higher proportion of patients within the targeted tacrolimus trough levels by day three after dose initiation achieving target concentration rapidly with fewer dose modifications [9, 13, 18]. In another study, it was observed that CYP3A4 and CYP3A5 genotypes could explain 56–59% variability in tacrolimus dose and clearance [25]. In a recent study, Tang et al, utilized nine machine learning tools to predict the tacrolimus stable dose, and found that all algorithms were equally effective in predicting tacrolimus therapeutic dose and further demonstrated that regression tree (RT) model was the best among the other tools in predicting the tacrolimus stable dose [46]. The major limitation of our study was the sample size. Even though we developed the algorithm to predict the bioavailability of tacrolimus, we need to validate in more number of cases for clinical utility of this algorithm for prediction of the therapeutic dose before the renal transplantation.

Furthermore, the presence of variant alleles at ABCB1 2677 and CYP3A5*3 was shown to increase the risk for post-transplant diabetes based on our MDR model, which coincides with the higher bioavailability of tacrolimus in this genotype combination. This corroborates with findings of Chitnis et al [47] who demonstrated higher dose normalized concentrations of tacrolimus in patients with post-transplant diabetes and the higher risk was observed in subjects with CYP3A5*3 polymorphism.

## Conclusion

In conclusion, consistent with the literature, this study also demonstrates that the CYP3A5*3 allele and ABCB1 polymorphisms are highly associated with tacrolimus bioavailability in renal transplant patients. In addition, this study confirms that the combination of multiple ABCB1 polymorphisms with CYP3A5 genotype as demonstrated by the ANN model has a stronger effect to calculate more precisely the initial tacrolimus dose to improve the therapy and to prevent the tacrolimus toxicity. The gene-gene interactions between ABCB1 and CYP3A5 also influence the risk for post-transplant diabetes.

## Supporting information

S1 Table Code file for artificial neural network based algorithm development. (XLS)

## Abbreviations

ABCB1: ATP-binding cassette sub-family B member 1
BK: BK virus
C0: tacrolimus trough concentrations
CMV: Cytomegalovirus
HCV: hepatitis C virus
HWE: Hardy–Weinberg equilibrium
log(Co/D: log dose-normalized concentration
MMF: mycophenolate mofetil
PCR-RFLP: polymerase chain reaction-restriction fragment length polymorphism

## References

[1] Wallemacq P, Armstrong VW, Brunet M, Haufroid V, Holt DW, Johnston A, et al Opportunities to Optimize Tacrolimus Therapy in Solid Organ Transplantation: Report of the European Consensus Conference. Therapeutic Drug Monitoring. 2009;31: 139–152. doi: 10.1097/FTD.0b013e318198d092 19177031 10.1097/FTD.0b013e318198d092

[2] Rodríguez-Perálvarez M, Germani G, Darius T, Lerut J, Tsochatzis E, Burroughs AK. Tacrolimus Trough Levels, Rejection and Renal Impairment in Liver Transplantation: A Systematic Review and Meta-Analysis. American Journal of Transplantation. 2012;12: 2797–2814. doi: 10.1111/j.1600-6143.2012.04140.x 22703529 10.1111/j.1600-6143.2012.04140.x

[3] Plosker GL, Foster RH. Tacrolimus: a further update of its pharmacology and therapeutic use in the management of organ transplantation. Drugs. 2000;59, 323–389. 10730553 10.2165/00003495-200059020-00021

[4] Kidokoro K, Satoh M, Nagasu H, Sakuta T, Kuwabara A, Yorimitsu D, et al Tacrolimus Induces Glomerular Injury via Endothelial Dysfunction Caused by Reactive Oxygen Species and Inflammatory Change. Kidney and Blood Pressure Research. 2012;35: 549–557. doi: 10.1159/000339494 22890154 10.1159/000339494

[5] Staatz C. Low tacrolimus concentrations and increased risk of early acute rejection in adult renal transplantation. Nephrology Dialysis Transplantation. 2001;16: 1905–1909. 10.1093/ndt/16.9.1905 11522877

[6] Undre N, Hooff JV, Christiaans M, Vanrenterghem Y, Donck J, Heeman U, et al Low systemic exposure to tacrolimus correlates with acute rejection. Transplantation Proceedings. 1999;31: 296–298. 10083114 10.1016/s0041-1345(98)01633-9

[7] Kuypers DRJ, Jonge HD, Naesens M, Lerut E, Verbeke K, Vanrenterghem Y. CYP3A5 and CYP3A4 but not MDR1 Single-nucleotide Polymorphisms Determine Long-term Tacrolimus Disposition and Drug-related Nephrotoxicity in Renal Recipients. Clinical Pharmacology & Therapeutics. 2007;82: 711–725. 17495880 10.1038/sj.clpt.6100216

[8] Gérard C, Stocco J, Hulin A, Blanchet B, Verstuyft C, Durand F, et al Determination of the Most Influential Sources of Variability in Tacrolimus Trough Blood Concentrations in Adult Liver Transplant Recipients: A Bottom-Up Approach. The AAPS Journal. 2014;16: 379–391. doi: 10.1208/s12248-014-9577-8 24526611 10.1208/s12248-014-9577-8 PMC4012056

[9] Kim I-W, Noh H, Ji E, Han N, Hong SH, Ha J, et al Identification of Factors Affecting Tacrolimus Level and 5-Year Clinical Outcome in Kidney Transplant Patients. Basic & Clinical Pharmacology & Toxicology. 2012;111, 217–223. 22469198 10.1111/j.1742-7843.2012.00892.x

[10] Cusinato DAC, Lacchini R, Romao EA, Moysés-Neto M, Coelho EB. Relationship of CYP3A5genotype andABCB1diplotype to tacrolimus disposition in Brazilian kidney transplant patients. British Journal of Clinical Pharmacology. 2014;78: 364–372. doi: 10.1111/bcp.12345 24528196 10.1111/bcp.12345 PMC4137828

[11] Haufroid V, Mourad M, Kerckhove VV, Wawrzyniak J, Meyer MD, Eddour DC, et al The effect of CYP3A5 and MDR1 (ABCB1) polymorphisms on cyclosporine and tacrolimus dose requirements and trough blood levels in stable renal transplant patients. Pharmacogenetics. 2004;14: 147–154. 15167702 10.1097/00008571-200403000-00002

[12] Tsuchiya N, Satoh S, Tada H, Li Z, Ohyama C, Sato K, et al Influence of CYP3A5 and MDR1 (ABCB1) Polymorphisms on the Pharmacokinetics of Tacrolimus in Renal Transplant Recipients. Transplantation. 2004;78: 1182–1187. 15502717 10.1097/01.tp.0000137789.58694.b4

[13] Provenzani A. Influence of CYP3A5 and ABCB1 gene polymorphisms and other factors on tacrolimus dosing in Caucasian liver and kidney transplant patients. International Journal of Molecular Medicine. 2011; 28, 1093–1102. doi: 10.3892/ijmm.2011.794 21922127 10.3892/ijmm.2011.794

[14] Kuehl P, Zhang J, Lin Y, Lamba J, Assem M, Schuetz J, et al Sequence diversity in CYP3A promoters and characterization of the genetic basis of polymorphic CYP3A5 expression. Nat Genet. 2001;27, 383–91. doi: 10.1038/86882 11279519 10.1038/86882

[15] Zuo X-C, Ng CM, Barrett JS, Luo A-J, Zhang B-K, Deng C-H, et al Effects of CYP3A4 and CYP3A5 polymorphisms on tacrolimus pharmacokinetics in Chinese adult renal transplant recipients. Pharmacogenetics and Genomics. 2013;23: 251–261. doi: 10.1097/FPC.0b013e32835fcbb6 23459029 10.1097/FPC.0b013e32835fcbb6

[16] Aouam K, Kolsi A, Kerkeni E, Fredj NB, Chaabane A, Monastiri K, et al Influence of combined CYP3A4 and CYP3A5 single-nucleotide polymorphisms on tacrolimus exposure in kidney transplant recipients: a study according to the post-transplant phase. Pharmacogenomics. 2015;16: 2045–2054. doi: 10.2217/pgs.15.138 26615671 10.2217/pgs.15.138

[17] Haufroid V, Mourad M, Kerckhove VV, Wawrzyniak J, Meyer MD, Eddour DC, et al The effect of CYP3A5 and MDR1 (ABCB1) polymorphisms on cyclosporine and tacrolimus dose requirements and trough blood levels in stable renal transplant patients. Pharmacogenetics. 2004;14: 147–154. 15167702 10.1097/00008571-200403000-00002

[18] Kim I-W, Moon YJ, Ji E, Kim KI, Han N, Kim SJ, et al Clinical and genetic factors affecting tacrolimus trough levels and drug-related outcomes in Korean kidney transplant recipients. European Journal of Clinical Pharmacology. 2011;68: 657–669. doi: 10.1007/s00228-011-1182-5 22183771 10.1007/s00228-011-1182-5

[19] Anglicheau D. Association of the Multidrug Resistance-1 Gene Single-Nucleotide Polymorphisms with the Tacrolimus Dose Requirements in Renal Transplant Recipients. Journal of the American Society of Nephrology. 2003;14: 1889–1896. 12819250 10.1097/01.asn.0000073901.94759.36

[20] Soria ML-M, Berga JK, Catalán SB, Payá JM, Mateu LP, Torres NJ. Genetic Polymorphisms and Individualized Tacrolimus Dosing. Transplantation Proceedings. 2010;42: 3031–3033. doi: 10.1016/j.transproceed.2010.08.001 20970601 10.1016/j.transproceed.2010.08.001

[21] Shi Y, Li Y, Tang J, Zhang J, Zou Y, Cai B, et al Influence of CYP3A4, CYP3A5 and MDR-1 polymorphisms on tacrolimus pharmacokinetics and early renal dysfunction in liver transplant recipients. Gene. 2013;512: 226–231. doi: 10.1016/j.gene.2012.10.048 23107770 10.1016/j.gene.2012.10.048

[22] Cho J-H, Yoon Y-D, Park J-Y, Song E-J, Choi J-Y, Yoon S-H, et al Impact of Cytochrome P450 3A and ATP-Binding Cassette Subfamily B Member 1 Polymorphisms on Tacrolimus Dose-Adjusted Trough Concentrations Among Korean Renal Transplant Recipients. Transplantation Proceedings. 2012;44: 109–114. doi: 10.1016/j.transproceed.2011.11.004 22310591 10.1016/j.transproceed.2011.11.004

[23] Fukudo M, Yano I, Yoshimura A, Masuda S, Uesugi M, Hosohata K, et al Impact of MDR1 and CYP3A5 on the oral clearance of tacrolimus and tacrolimus-related renal dysfunction in adult living-donor liver transplant patients. Pharmacogenetics and Genomics. 2008;18: 413–423. doi: 10.1097/FPC.0b013e3282f9ac01 18408564 10.1097/FPC.0b013e3282f9ac01

[24] Provenzani A, Notarbartolo M, Labbozzetta M, Poma P, Biondi F, Sanguedolce R, et al The effect of CYP3A5 and ABCB1 single nucleotide polymorphisms on tacrolimus dose requirements in Caucasian liver transplant patients. Ann Transplant. 2009;14: 23–31. 19289993

[25] Staatz CE, Goodman LK, Tett SE. Effect of CYP3A and ABCB1 Single Nucleotide Polymorphisms on the Pharmacokinetics and Pharmacodynamics of Calcineurin Inhibitors: Part I. Clinical Pharmacokinetics. 2010;49: 141–175. doi: 10.2165/11317350-000000000-00000 20170205 10.2165/11317350-000000000-00000

[26] Jonge HD, Loor HD, Verbeke K, Vanrenterghem Y, Kuypers DR. In Vivo CYP3A4 Activity, CYP3A5 Genotype, and Hematocrit Predict Tacrolimus Dose Requirements and Clearance in Renal Transplant Patients. Clinical Pharmacology & Therapeutics. 2012;92: 366–375. 22871995 10.1038/clpt.2012.109

[27] Thervet E, Loriot MA, Barbier S, Buchler M, Ficheux M, Choukroun G, et al Optimization of Initial Tacrolimus Dose Using Pharmacogenetic Testing. Clinical Pharmacology & Therapeutics. 2010;87, 721–726. 20393454 10.1038/clpt.2010.17

[28] Li J-L, Wang X-D, Chen S-Y, Liu L-S, Fu Q, Chen X, et al Effects of diltiazem on pharmacokinetics of tacrolimus in relation to CYP3A5 genotype status in renal recipients: from retrospective to prospective. The Pharmacogenomics Journal. 2010;11: 300–306. doi: 10.1038/tpj.2010.42 20514078 10.1038/tpj.2010.42

[29] Passey C, Birnbaum AK, Brundage RC, Schladt DP, Oetting WS, Leduc RE, et al Validation of tacrolimus equation to predict troughs using genetic and clinical factors. Pharmacogenomics. 2012;13: 1141–1147. doi: 10.2217/pgs.12.98 22909204 10.2217/pgs.12.98 PMC3579500

[30] Passey C, Birnbaum AK, Brundage RC, Oetting WS, Israni AK, Jacobson PA. Dosing equation for tacrolimus using genetic variants and clinical factors. British Journal of Clinical Pharmacology. 2011;72: 948–957. doi: 10.1111/j.1365-2125.2011.04039.x 21671989 10.1111/j.1365-2125.2011.04039.x PMC3244642

[31] Li L, Li C-J, Zheng L, Zhang Y-J, Jiang H-X, Si-Tu B, et al Tacrolimus dosing in Chinese renal transplant recipients: a population-based pharmacogenetics study. European Journal of Clinical Pharmacology. 2011;67: 787–795. doi: 10.1007/s00228-011-1010-y 21331500 10.1007/s00228-011-1010-y

[32] Wang P, Mao Y, Razo J, Zhou X, Wong ST, Patel S, et al Using genetic and clinical factors to predict tacrolimus dose in renal transplant recipients. Pharmacogenomics. 2010;11: 1389–1402. doi: 10.2217/pgs.10.105 21047202 10.2217/pgs.10.105

[33] Tahara T, Shibata T, Yamashita H, Hirata I, Arisawa T. Influence of MDR1 Polymorphism on H. pylori-Related Chronic Gastritis. Digestive Diseases and Sciences. 2010;56: 103–108. doi: 10.1007/s10620-010-1251-0 20464493 10.1007/s10620-010-1251-0

[34] Omar MS. & Hughes J. Distribution of the single nucleotide polymorphism C3435T of MDR1 gene among people in Western Australia, Australia. International Journal of Pharmacy & Pharmaceutical Sciences. 2013;5: 470–473

[35] Borobia AM, Romero I, Jimenez C, Gil F, Ramirez E, Gracia RD, et al Trough Tacrolimus Concentrations in the First Week After Kidney Transplantation Are Related to Acute Rejection. Therapeutic Drug Monitoring. 2009;31: 436–442. doi: 10.1097/FTD.0b013e3181a8f02a 19494792 10.1097/FTD.0b013e3181a8f02a

[36] Renders L, Frisman M, Ufer M, Mosyagin I, Haenisch S, Ott U, et al CYP3A5 Genotype Markedly Influences the Pharmacokinetics of Tacrolimus and Sirolimus in Kidney Transplant Recipients. Clinical Pharmacology & Therapeutics. 2006;81: 228–234. 17192769 10.1038/sj.clpt.6100039

[37] Macphee IA, Fredericks S, Mohamed M, Moreton M, Carter ND, Johnston A, et al Tacrolimus Pharmacogenetics: The CYP3A5*1 Allele Predicts Low Dose-Normalized Tacrolimus Blood Concentrations in Whites and South Asians. Transplantation. 2005;79: 499–502. 15729180 10.1097/01.tp.0000151766.73249.12

[38] Goto M, Masuda S, Kiuchi T, Ogura Y, Oike F, Okuda M, et al CYP3A5*1-carrying graft liver reduces the concentration/oral dose ratio of tacrolimus in recipients of living-donor liver transplantation. Pharmacogenetics. 2004;14: 471–478. 15226679 10.1097/01.fpc.0000114747.08559.49

[39] Hesselink DA, Schaik RHV, Agteren MV, Fijter JWD, Hartmann A, Zeier M, et al CYP3A5 genotype is not associated with a higher risk of acute rejection in tacrolimus-treated renal transplant recipients. Pharmacogenetics and Genomics. 2008;18: 339–348. doi: 10.1097/FPC.0b013e3282f75f88 18334918 10.1097/FPC.0b013e3282f75f88

[40] Tang K, Ngoi S-M, Gwee P-C, Chua JMZ, Lee EJD, Chong SS, et al Distinct haplotype profiles and strong linkage disequilibrium at the MDR1 multidrug transporter gene locus in three ethnic Asian populations. Pharmacogenetics. 2002;12: 437–450. 12172212 10.1097/00008571-200208000-00004

[41] Singh R, Srivastava A, Kapoor R, Sharma RK, Mittal RD. Impact of CYP3A5 and CYP3A4 gene polymorphisms on dose requirement of calcineurin inhibitors, cyclosporine and tacrolimus, in renal allograft recipients of North India. Naunyn-Schmiedeberg's Archives of Pharmacology. 2009;380: 169–177. doi: 10.1007/s00210-009-0415-y 19343327 10.1007/s00210-009-0415-y

[42] Singh R, Srivastava A, Kapoor R, Mittal RD. Do Drug Transporter (ABCB1) SNPs Influence Cyclosporine and Tacrolimus Dose Requirements and Renal Allograft Outcome in the Posttransplantation Period? The Journal of Clinical Pharmacology. 2011;51: 603–615. doi: 10.1177/0091270010370704 20571034 10.1177/0091270010370704

[43] Chandel N, Aggarwal PK, Minz M, Sakhuja V, Kohli KK, Jha V. CYP3A5*1/*3 genotype influences the blood concentration of tacrolimus in response to metabolic inhibition by ketoconazole. Pharmacogenetics and Genomics. 2009;19: 458–463. doi: 10.1097/FPC.0b013e32832bd085 19384264 10.1097/FPC.0b013e32832bd085

[44] Pavani A, Naushad SM, Rupasree Y, Kumar TR, Malempati AR, Pinjala RK, et al Optimization of warfarin dose by population-specific pharmacogenomic algorithm. The Pharmacogenomics Journal. 2011;12: 306–311. doi: 10.1038/tpj.2011.4 21358752 10.1038/tpj.2011.4

[45] Pavani A, Naushad SM, Kumar RM, Srinath M, Malempati AR, Kutala VK. Artificial neural network-based pharmacogenomic algorithm for warfarin dose optimization. Pharmacogenomics. 2016;17: 121–131. doi: 10.2217/pgs.15.161 26666467 10.2217/pgs.15.161

[46] Tang J, Liu R, Zhang Y-L, Liu M-Z, Hu Y-F, Shao M-J, et al Application of Machine-Learning Models to Predict Tacrolimus Stable Dose in Renal Transplant Recipients. Scientific Reports. 2017;7: 42192 doi: 10.1038/srep42192 28176850 10.1038/srep42192 PMC5296901

[47] Chitnis SD, Ogasawara K, Schniedewind B, Gohh RY, Christians U, Akhlaghi F. Concentration of tacrolimus and major metabolites in kidney transplant recipients as a function of diabetes mellitus and cytochrome P450 3A gene polymorphism. Xenobiotica. 2013;43: 641–649. doi: 10.3109/00498254.2012.752118 23278282 10.3109/00498254.2012.752118 PMC4116556

